# Dentistry in the Army

**Published:** 1919

**Authors:** 


***Dentistry in the Army.*** By ALONZO MILTON NODINE. *American Journal of Surgery*, July 1918.

The author gives a detailed account of his journey to France and speaks of the work done at the front by the Dental Service. The condition of the teeth of the wounded arriving at a hospital is, as a rule, bad. Nineteen out of every twenty men need fillings, all need to have their teeth scaled and cleaned, and about fifty per cent. have abscesses.

The number of men suffering from Riggs' Disease is comparatively small. Under favorable conditions, an oral and dental service in a hospital may be able to improve the facial appearance of men with face wounds. It may also help those who need all the nutrition they can get to chew and therefore to digest properly. An effective masticating apparatus is a most important factor in the recovery of the wounded.

Again, diseased and abscessed teeth are a source of poison. Hidden abscesses and dead teeth furnish micro-organisms and toxins that neutralize the constructive forces of the body. The constant ingestion of food, mixed with pus and the discharges of diseased teeth and gums, affects the digestion and adds another source of infection for the system to overcome.

The author insists upon the necessity for keeping the masticating apparatus in a fit condition, and cites a number of cases illustrating the results that may be secured.

